# Risk factors associated with unsuccessful tuberculosis treatment outcomes in Hunan Province, China

**DOI:** 10.1111/tmi.13720

**Published:** 2022-02-06

**Authors:** Beth Gilmour, Zuhui Xu, Liqiong Bai, Kefyalew Addis Alene, Archie C. A. Clements

**Affiliations:** ^1^ Faculty of Health Sciences Curtin University Bentley WA Australia; ^2^ Xiangya School of Public Health Central South University Changsha China; ^3^ TB Control Institute of Hunan Province Changsha China; ^4^ Telethon Kids Institute Nedlands WA Australia

**Keywords:** China, risk factors, treatment outcome, tuberculosis

## Abstract

**Objectives:**

Globally, China has the third highest number of tuberculosis (TB) cases despite high rates (85.6%) of effective treatment coverage. Identifying risk factors associated with unsuccessful treatment outcomes is an important component of maximising the efficacy of TB control programmes.

**Methods:**

Retrospective cohort study to evaluate the outcomes of 306,860 drug‐susceptible TB patients who underwent treatment in Hunan Province, China between 2013 and 2018. Univariable and multivariable logistic regression models were used to identify factors associated with unsuccessful TB treatment outcomes.

**Results:**

A successful treatment outcome was recorded for 98.6% of patients, defined as the sum of patients who were cured (36.2%) and completed treatment (62.4%). An unsuccessful treatment outcome was recorded for 1.8% of patients, defined as the sum of treatment failure (1.1%), deaths (0.5%) and lost to follow up (0.2%). The odds of an unsuccessful treatment outcome showed an increasing trend in more recent years of registration (2018 adjusted odds ratio (AOR): 1.43; 95% Confidence Interval (CI) 1.31, 1.57 relative to 2013). Other significant risk factors were male sex (AOR: 1.17; 95% CI 1.10, 1.25); increasing age (AOR:1.02 per year increase; 95% CI 1.02,1.02); being severely ill (AOR: 1.50; 95% CI 1.33, 1.70); having a history of TB treatment (AOR: 2.93; 95% CI 2.69, 3.20); not being under systematic management (AOR: 16.10 (14.49, 17.88) and treatment regimens that differed from full course management.

**Conclusions:**

The increasing likelihood of an unsuccessful treatment outcome over time necessitates the need for further research.

## INTRODUCTION

Throughout history *Mycobacterium tuberculosis* (MTB), the pathogen responsible for tuberculosis (TB), is thought to have claimed more lives than any other microorganism [1]. With an estimated 1.4 million lives lost to the disease in 2019, TB continues to be one of the leading infectious causes of death globally [2]. Tuberculosis is associated with poverty and it fuels the cycle of deprivation and vulnerability [3].

Tuberculosis can be cured, but if left untreated the mortality rate is high, with 10‐year case fatality rates ranging between 54 and 86% in human immunodeficiency virus (HIV) negative patients [4]. For drug‐susceptible TB, a 6‐month treatment regime containing four first‐line antibiotics (i.e. isoniazid, rifampicin, ethambutol and pyrazinamide) is recommended, which has an 85% success rate [3]. Successful treatment is key to curing the disease, preventing transmission of infection and preventing the development of drug resistance [3]. Drug‐resistant TB is an escalating global health security threat [2], projected to cost the world US$ 16.7 trillion by 2050 [5].

Previous studies have found a number of factors to be associated with unsuccessful TB treatment outcomes, including positive HIV status, male sex, ethnicity, low body mass index (BMI), substance abuse, other co‐morbidities, previous treatment, drug resistance, low level of education, lack of knowledge on treatment duration and the importance of treatment completion, household income, the requirement for hospitalisation during treatment, side effects of medication, improved symptoms resulting in the cessation of therapy, lack of family support and unsupervised treatment administration [6, 7, 8, 9, 10, 11, 12, 13. The factors relating to unsuccessful treatment outcomes need to be understood and addressed to maximise the efficacy of TB control programmes and prevent escalating drug resistance.

In 2014, the World Health Assembly adopted the *End TB Strategy*, which is integral to Sustainable Development Goal 3.3 that aims to end the TB epidemic by 2030 [14, 15. By 2030, the End TB Strategy aims to reduce TB deaths by 90%, reduce TB incidence by 80% and eliminate catastrophic costs faced by TB households [15].

In terms of 2019 TB cases numbers, China ranks third with 8.4% of the global total [3], despite effective treatment coverage being estimated at >85.6% [16]. In 2019, China had the second‐greatest burden (14%) of multidrug‐resistant TB (MDR‐TB), which was estimated to occur in 7.1% of new and 23% of previously treated cases [3]. To address the burden of disease, China has initiated a National Tuberculosis Control Programme (NTP) based on the Directly Observed Treatment Short‐course (DOTS) strategy recommended by WHO [17]. Although the NTP aims to provide TB diagnosis and treatment services free of charge, patients often face significant out of pocket expenses and financial hardship [18].

Hunan province, located in south‐central China, carries a high burden of TB despite significant investments that have been made by the Hunan government to combat the disease [19, 20, 21. An understanding of the risk factors associated with unsuccessful treatment outcomes in province‐specific TB patient populations could help reduce the burden of disease by informing targeted interventions, for example, systematic drug supervision, sex‐specific TB education/messaging. Few of the studies on risk factors associated with unsuccessful TB outcomes have been conducted in China. To our knowledge, only one study has evaluated treatment default and mortality in TB patients that were registered in Hunan between 2005 and 2006 [22]. Our study aimed to evaluate the rate of treatment success and the risk factors associated with unsuccessful treatment outcomes among drug‐susceptible TB (DS‐TB) patients in Hunan Province who were undergoing treatment between 2013 and 2018.

## METHODS

### Study design and data sources

This is a retrospective cohort study conducted on patients undergoing treatment for pulmonary and extrapulmonary DS‐TB in Hunan Province, China between 2013 and 2018 inclusive. Within China, TB is a category II notifiable disease and health professionals are responsible for the collection and entry of data from notified patients into an Internet‐based TB management information system [23]. Within Hunan, the TB management information system is managed by the Tuberculosis Control Institute of Hunan Province (TBCIH), which provided access to the data for this study. Clinical data relating to the date of treatment commencement, date of treatment completion, treatment outcome and type of treatment management were available, as were demographic data such as ethnicity, age, sex, occupation and residential address.

### Definitions

We used the WHO definitions of treatment outcomes: [24]

**Table:** 

Outcome	Definition
Cured	A pulmonary TB patient with bacteriologically confirmed TB at the beginning of treatment who was smear‐ or culture‐negative in the last month of treatment and on at least one previous occasion.
Treatment completed	A TB patient who completed treatment without evidence of failure BUT with no record to show that sputum smear or culture results in the last month of treatment and on at least one previous occasion were negative, either because tests were not done or because results are unavailable.
Treatment failed	A TB patient whose sputum smear or culture is positive at month five or later during treatment.
Died	A TB patient who dies for any reason before starting or during the course of treatment.
Lost to follow up	A TB patient who did not start treatment or whose treatment was interrupted for two consecutive months or more.
Not evaluated	A TB patient for whom no treatment outcome is assigned. This includes cases ‘transferred out’ to another treatment unit as well as cases for whom the treatment outcome is unknown to the reporting unit.
Treatment success	The sum of cured and treatment completed

To dichotomise data into successful and unsuccessful treatment outcomes, treatment success was classified as ‘cured’ plus ‘treatment completed’ and an unsuccessful outcome as the sum of ‘treatment failed’, ‘died’ and ‘lost to follow‐up’. Definitions pertaining to the other demographic descriptors/variables analysed are detailed in Table 1.

**TABLE 1:** Definitions of the variables included and relating to our study

Variable	Definition
Residential address	
Local	Patients who reside in local counties
Intra‐provincial	Patients who reside in other counties within the province
Inter‐provincial	Patients who reside in provinces other than Hunan
Foreign nationality	Patients who reside in other countries
Registration category	
New patient	PTB patients who have never taken anti‐TB drugs, or who have been receiving irregular treatment for less than one month
Relapse	PTB patients with a history of disease, who complete a full course of chemotherapy and appear cured according to symptoms, but who return a smear positive sputum sample
Return after default	PTB patients who receive chemotherapy for ≥1 month but discontinue therapy for ≥2 months and then return for treatment
Initial treatment failed	New sputum smear positive PTB patients with positive sputum smear microscopy results at the end of the 5th month or after completion of therapy; and sputum smear negative PTB patients with a positive smear result for any sputum sample
Chronic patient	Positive sputum examination results after several episodes of irregular therapy
TB diagnosis results	
Etiological examination negative	TB cases confirmed on basis of symptoms
Smear positive	Positive Acid‐Fast Bacillus test
Extrapulmonary TB	TB identified in organs other than the lungs
Culture positive	Positive sputum culture
Molecular biology positive	TB confirmed on basis of molecular diagnosis
Severely ill	Patients with miliary TB, cavities, TB empyema or serious damage to one or more organs caused by TB infection.
Drug resistance pattern	
Drug susceptible TB	*M*. *tuberculosis* that is susceptible to first line antibiotics (isoniazid, rifampin, ethambutol, and pyrazinamide)
MDR‐TB	*M*. *tuberculosis* resistant to isoniazid and rifampicin
Mono‐resistant TB	*M*. *tuberculosis* resistant to a single first line antibiotic
History of TB treatment	
No (Initial treatment)	a patient who has never taken anti‐TB drugs; or a patient receiving standardized TB treatment but who has not completed the full course of treatment; or a patient receiving irregular TB treatment for less than one month.
Yes (Retreatment)	a patient receiving irregular anti‐TB drugs for one month or longer; or initial treatment failure and relapse
TB treatment outcomes [50]	
Treatment completed	A TB patient who completed treatment without evidence of failure BUT with no record to show that sputum smear or culture results in the last month of treatment and on at least one previous occasion were negative, either because tests were not done or because results are unavailable.
Cured	A pulmonary TB patient with bacteriologically confirmed TB at the beginning of treatment who was smear‐ or culture‐negative in the last month of treatment and on at least one previous occasion.
Treatment failure	A TB patient whose sputum smear or culture is positive at month five or later during treatment.
Death	A TB patient who dies for any reason before starting or during the course of treatment.
Lost to follow‐up	A TB patient who did not start treatment or whose treatment was interrupted for two consecutive months or more
Successful treatment outcome	The sum of cured and treatment completed
Unsuccessful treatment outcome	The sum of treatment failure, death and lost to follow up
Treatment management	
Full process supervision	Patients take all TB medications under the direct observation of a medication supervisor during the full course of treatment.
Intensive phase supervision	Patients take all TB medications under the direct observation of a medication supervisor during the intensive phase. Full‐course management is conducted during the continuation phase.
Full course management	Comprehensive management is conducted during the full course of TB treatment to ensure medications are taken regularly. This includes health education; regular drug collection; cross checking, tracing, and patient visits in the event of failure to collect drugs/visit the clinic.
Self‐administered medication	Health education is provided on standardized chemotherapy and patients self‐medicate
Systematic management	A registered PTB patient who has accepted timely sputum examinations, medication supervision and regular treatment
China recognizes 56 ethnic classifications comprising the Han majority and 55 minority groups [51]. For this study, data were analysed for Han majority and Tujia, Miao, Dong, Yao, Bai, Mongolian and ‘other’ ethnic minority groups. The ‘other’ ethnic minority grouping comprises the summation of ethnic minority groups which constitute <0.1% of the patient population. The ‘other’ group was represented by Buyi, Dai, Gelao, Hani, Hui, Jingpo, Kazakh, Kirgiz, Korean, Lahu, Li, Lisu, Manchu, Salar, She, Tibetan, Tu, Uighur, Wa, Yao, Yi, and Zhuang ethnic minorities.	

Note

Effective treatment coverage = ‘an indicator that combines treatment coverage and the treatment success rate to estimate the proportion of TB cases that are detected and successfully treated’ [52].

### Statistical analysis

Data were translated from Mandarin to English, cleaned, checked for completeness and entered into STATA version 16.1 (StataCorp, College Station, TX) for analysis. Cross‐tabulation was used to verify data completeness. The following data were excluded from the original data set prior to analysis: patients who were still on treatment and those who were transferred out, for example, diagnosis changed, HIV +ve, MDR‐TB. The treatment outcomes of patients transferred out were not recorded in the TBCIH database.

Descriptive statistics were used to summarise data and illustrate characteristics of the study population. Univariable logistic regression models were performed and crude odds ratios (COR) with 95% confidence intervals (CI) were reported. Multicollinearity between independent variables was assessed by variance inflation factors (VIF) and variables with a high degree of association with other independent variables (i.e. VIF >5) were excluded from the final models.

All independent variables with a VIF <5 were included in multivariable logistic regression models and adjusted odds ratios with 95% CIs used to determine the strength of association between the dependent and independent variables. In the multivariable regression analysis, variables with a *p*‐value <0.05 were considered significantly associated with an unsuccessful treatment outcome.

### Ethical approval

Ethics approval was obtained from Curtin University (HRE2019‐0581) and permission to access the data was obtained from TBCIH. As this study used secondary and routinely collected clinical data, informed consent was not obtained from the study participants. Medical records were anonymised by TBCIH to maintain patient confidentiality.

## RESULTS

**FIGURE 1:**
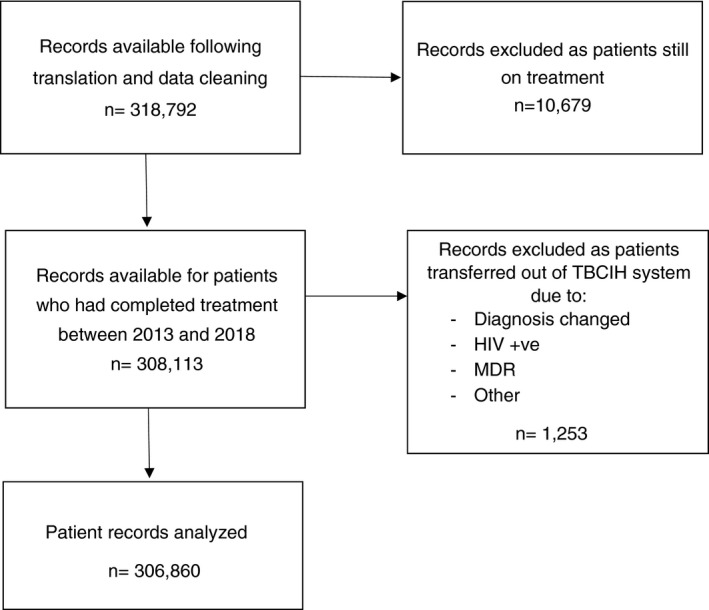
Flowchart of patient record selection process

Figure 1 details the patient record selection process: 318,792 records were available after translation and data cleaning. The data set included patients on treatment between 2013 and 2018; we were in receipt of this in 2018 and so some patients were yet to complete their course of treatment and were excluded (*n* = 10,679). Of the patients that had completed treatment (*n* = 308,113), records for those transferred out (*n* = 1,253) were excluded, as their treatment outcomes were not recorded on the TBCIH database.

### Socio‐demographic and clinical characteristics of the TB patients

The sociodemographic characteristics of the final patient cohort (*n* = 306,860) are detailed in Table 2. The mean age of the patient population was 51.6 years (SD 17.6), the majority was male (72.6%), employed in agriculture (78.3%) and new patients (95.9%).

**TABLE 2:** Sociodemographic characteristics of TB patients registered for treatment in Hunan Province, China, 2013–2018

Variable	Number	Percent
Sex		
Male	222,783	72.60
Female	84,077	27.40

Mean age 51.6 years (SD 17.6)		
Occupation		
Agriculture	240,235	78.29
Housekeeping, childcare, retired, un‐employed	29,144	9.50
Education^a^	10,341	3.37
Commercial services/civil servant	7,479	2.44
Migrant worker	2,531	0.82
Healthcare	962	0.31
Hospitality	586	0.19
Other	15,582	5.08
Ethnicity		
Han	277,813	90.54
Tujia	13,254	4.32
Miao	8,168	2.66
Dong	3,888	1.27
Yao	2,621	0.85
Bai	496	0.16
Mongolian	337	0.11
Other^b^	279	0.09
Residential address		
Local	298,844	97.39
Intra‐provincial	5,896	1.92
Inter‐provincial	2,071	0.67
Foreign nationality	49	0.02
Registration category^c^		
New patient	294,355	95.92
Relapse	11,210	3.65
Return after default	328	0.11
Initial treatment failed	246	0.08
Chronic patient	120	0.04
TB diagnosis results		
Etiological examination negative	182,343	59.42
Smear positive	117,491	38.29
Extrapulmonary TB	5,031	1.64
Only culture positive	1,154	0.38
Only molecular biology positive	637	0.21
No etiological results	180	0.06
Only pathologically positive	24	0.01
Severely ill		
No	295,172	96.19
Yes	11,688	3.81
History of TB treatment		
No	294,439	95.95
Yes	12,421	4.05

Median treatment time 184 days (IQR 182, 188)		
TB treatment outcomes		
Treatment completed	190,372	62.04
Cured	111,089	36.20
Treatment failure	3,317	1.08
Death	1,426	0.46
Lost to follow‐up	656	0.21
Treatment management		
Full process supervision	178,325	58.24
Intensive phase supervision	83,506	27.27
Full course management	41,381	13.52
Self‐administered medication	2,954	0.96
Successful TB treatment outcome	301,461	98.24
Unsuccessful TB treatment outcome	5,399	1.76
Systematic management		
Yes	303,924	99.04
No	2,936	0.96

^a^ Education includes both teachers and students.

^b^ Other are represented by 21 separately defined ethnic groups. NB ethnicity data are not available for four patients.

^c^ Patient registration category not available for 601 patients.

### Unsuccessful TB treatment outcomes

A successful treatment outcome was recorded for 98.24% of the patient population (treatment completed 62.04% and cured 36.20%). An unsuccessful treatment outcome was recorded for 1.76% of the patient population (treatment failure 1.08%, death 0.46% and lost to follow up 0.21%).

### Risk factors associated with an unsuccessful TB treatment outcome

Table 3 shows results of univariable and multivariable logistic regression models and factors associated with an unsuccessful treatment outcome. In the univariable analysis, demographic factors such as male sex, increasing age, occupation (i.e. agriculture housekeeping, childcare, retired and un‐employed) and year of enrolment; and clinical factors such as severe illness, non‐systematic management and supervision process were significantly associated with unsuccessful TB treatment outcomes.

**TABLE 3:** Univariable and multivariable logistic regression model results assessing factors associated with an unsuccessful TB treatment outcome

Risk factor for n TB treatment outcome	TB treatment outcome		Univariable estimate	Univariable *p* value	Multivariable estimate	Multivariable *p* value
	No Successful (%)	No. Unsuccessful (%)				
Ethnicity						
Han	272,912 (98.24)	4,91 (1.76)	1.00		1.00	
Tujia	13,031 (98.32)	223 (1.68)	0.95 (0.83, 1.09)	0.485	0.93 (0.81, 1.07)	0.337
Miao	8,018 (98.16)	150 (1.84)	1.04 (0.88, 1.23)	0.625	1.08 (0.92, 1.28)	0.334
Dong	3,816 (98.15)	72 (1.85)	1.05 (0.83, 1.33)	0.680	1.20 (0.95, 1.53)	0.130
Yao	2,583 (98.55)	38 (1.45)	0.82 (0.59, 1.13)	0.224	0.69 (0.49, 0.97)	0.033
Bai	492 (99.19)	4 (0.81)	0.45 (0.17, 1.21)	0.115	0.42 (0.16, 1.13)	0.083
Mongolian	331 (98.22)	6 (1.78)	1.01 (0.45, 2.26)	0.982	1.11 (0.49, 2.49)	0.803
Other*	274 (98.21)	5 (1.79)	1.02 (0.42, 2.46)	0.972	1.22 (0.50, 2.98)	0.660
Sex						
Female	82,847 (98.54)	1,230 (1.46)	1.00		1.00	
Male	218,614 (98.13)	4,169 (1.87)	1.28 (1.20, 1.37)	<0.0001	1.17 (1.10, 1.25)	<0.0001
Age (mean, years)	51.4	58.4	1.02 (1.02, 1.03)	<0.0001	1.02 (1.02, 1.02)	<0.0001
Occupation						
Comm services/civil servant	7,384 (98.73)	95 (1.27)	1.00		1.00	
Agriculture	235,774 (98.14)	4,461 (1.86)	1.47 (1.20, 1.80)	<0.0001	1.04 (0.84, 1.29)	0.707
At home^a^	28,618 (98.20)	526 (1.80)	1.43 (1.15, 1.78)	0.001	0.99 (0.79, 1.24)	0.917
Education	10,263 (99.25)	78 (0.75)	0.59 (0.44, 0.80)	0.001	0.98 (0.72, 1.33)	0.878
Migrant worker	2,506 (99.01)	25 (0.99)	0.78 (0.50, 1.21)	0.260	0.74 (0.48, 1.17)	0.196
Healthcare	956 (99.38)	6 (0.62)	0.49 (0.21, 1.12)	0.089	0.57 (0.25, 1.31)	0.186
Hospitality	575 (98.12)	11 (1.88)	1.49 (0.79, 2.79)	0.217	1.74 (0.91, 3.31)	0.093
Other	15,385 (98.74)	197 (1.26)	1.00 (0.78, 1.27)	0.970	0.91 (0.71, 1.17)	0.473
Year						
2013	53,660 (98.38)	886 (1.62)	1.00		1.00	
2014	53,501 (98.37)	886 (1.63)	1.00 (0.91, 1.10)	0.951	1.01 (0.92, 1.12)	0.768
2015	53,051 (98.47)	824 (1.53)	0.94 (0.85, 1.04)	0.210	0.97 (0.88, 1.07)	0.526
2016	47,634 (98.26)	842 (1.74)	1.07 (0.97, 1.18)	0.160	1.12 (1.02, 1.23)	0.022
2017	47,407 (98.20)	869 (1.80)	1.11 (1.01, 1.22)	0.030	1.12 (1.02, 1.23)	0.022
2018	46,208 (97.69)	1,092 (2.31)	1.43 (1.31, 1.57)	<0.0001	1.43 (1.31, 1.57)	<0.0001
Residential address						
Local	293,564 (98.23)	5,280 (1.77)	1.00		1.00	
Intra‐provincial	5,808 (98.51)	88 (1.49)	0.84 (0.68, 1.04)	0.113	0.92 (0.74, 1.15)	0.467
Inter‐provincial	2,040 (98.50)	31 (1.5)	0.84 (0.59, 1.21)	0.353	1.16 (0.80, 1.66)	0.434
Foreign nationality	49 (100)	–	–	–	–	–
Severely ill						
No	290, 085 (98.28)	5,087 (1.72)	1.00		1.00	
Yes	11,376 (97.33)	312 (2.67)	1.56 (1.39, 1.76)	<0.0001	1.50 (1.33, 1.70)	<0.0001
History of TB treatment						
No	289,756 (98.41)	4,683 (1.59)	1.00		1.00	
Yes	11,705 (94.24)	716 (5.76)	3.78 (3.49, 4.10)	<0.0001	2.93 (2.69, 3.20)	<0.0001
Treatment management						
Full course management	40,860 (98.74)	521 (1.26)	1.00		1.00	
Full process supervision	174,865 (98.06)	3,460 (1.94)	1.55 (1.41, 1.70)	<0.0001	1.51 (1.37, 1.66)	<0.0001
Intensive phase supervision	82,194 (98.43)	1,312 (1.57)	1.25 (1.13, 1.39)	<0.0001	1.39 (1.26, 1.55)	<0.0001
Self‐administered medication	2,882 (97.56)	72 (2.44)	1.96 (1.53, 2.51)	<0.0001	1.98 (1.53, 2.55)	<0.0001
Systematic management						
Yes	299,075 (98.40)	4,849 (1.60)	1.00		1.00	
No	2,386 (81.27)	550 (18.73)	14.21 (12.90, 15.66)	<0.0001	16.10 (14.49, 17.88)	<0.0001

Note

‘Registration category’ has been excluded from the regression analysis due to multicollinearity with the variable ‘History of TB treatment’.

Patients with a residential address of ‘foreign nationality’ have been excluded from the regression analysis as no patients within this category had an unsuccessful treatment outcome.

*Other are represented by: Buyi, Dai, Gelao, Hani, Hui, Jingpo, Kazakh, Kirgiz, Korean, Lahu, Li, Lisu,Manchu, Salar, She, Tibetan, Tu, Uighur, Wa, Yao, Yi, and Zhuang ethnic groups.

^a^ At home = housekeeping, childcare, retired, un‐employed.

**FIGURE 2:**
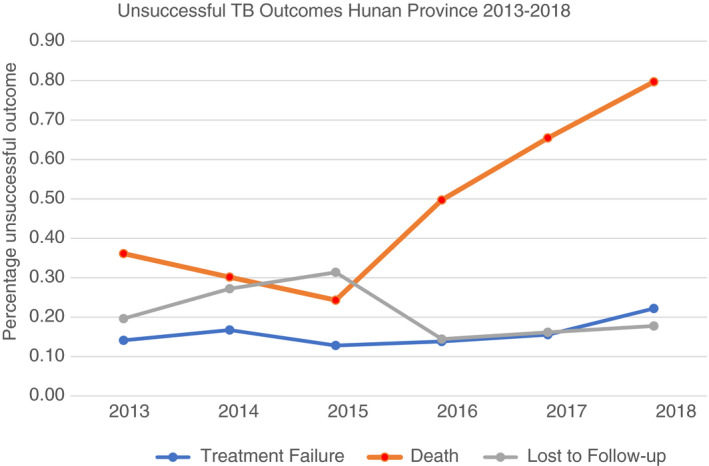
Unsuccessful TB treatment outcomes Hunan Province 2013–2018

In the final multivariable analysis, male sex (AOR:1.17; 95% CI 1.10, 1.25), increasing age (AOR:1.02 per year increase; 95% CI 1.02, 1.02) and being severely ill (AOR: 1.50; 95% CI 1.33, 1.70) were significant risk factors for unsuccessful treatment outcomes. The odds of an unsuccessful treatment outcome were greater where a patient was not systematically managed (AOR: 16.10; 95% CI 14.49, 17.88) and when they were under full process supervision (AOR: 1.51 (95% CI 1.37, 1.66); intensive phase supervision (AOR: 1.39; 95% CI 1.26, 1.55) or self ‐administered medication (AOR: 1.98; 95% CI 1.53, 2.55) relative to full course management. Registration in the years 2016–2018 was also associated with an unsuccessful treatment outcome relative to 2013 (2016 AOR: 1.12; 95% CI 1.02, 1.23; 2017 AOR:1.12; 95% CI 1.02, 1.23 and 2018 AOR:1.43; 95% CI 1.31, 1.57). An evaluation of the factors contributing to unsuccessful treatment outcome over time shows an increasing mortality rate in the later years of patient registration (Figure 2).

## DISCUSSION

This study found a treatment success rate of 98.24% amongst DS‐TB patients undergoing therapy between 2013 and 2018 in Hunan Province, China. This figure is higher than the 94% success rate reported for new cases across China in 2018 [25], and the WHO target of 85% [26]. It is noted, however, that the exclusion of patients who were transferred out, may have increased the reported success rate.

Our study found the likelihood of unsuccessful treatment outcome to be higher in the last three years of patient enrolment, with an increasing trend over time. Within the variables that contribute to an unsuccessful treatment outcome, there is an increasing trend in the mortality rate over recent years. Modelling studies have suggested that an increasing trend of unsuccessful TB treatment outcomes may be related to the increasing prevalence of MDR‐TB and the relatively low rate of MDR detection and treatment in China [3, 27, 28. Further research is required to elucidate whether the increasing trend in mortality is related to an increasing prevalence of MDR‐TB or whether it relates to other factors such as disease severity, age or co‐morbidities. Among the causes for TB treatment default, economic hardship is cited as one of the most common reasons [7, 9, 18, 27 with 2019 global figures estimating that 44% of people with DS‐TB and 80% of people with MDR‐TB face catastrophic costs [3]. Although the Chinese Action Plan to Stop TB (2019–2022) aims to provide drug susceptibility testing (DST) to 90% of bacteriologically confirmed cases by 2022 [27], the cost implications of the additional resources required to detect and treat MDR are not fully covered by Chinese health insurance schemes [18, 27, 29. Interventions that identify and help patients facing catastrophic costs maybe an effective way of improving the efficacy of TB programme outcomes.

Within the Hunan study population, male sex and increasing age were associated with increased odds of unsuccessful treatment outcome. The finding supports sex specific TB education and messaging. Gender differences in TB treatment outcomes remain inconsistent, although a number of studies support our finding [30, 31, 32, 33. Possible explanations for sex disparities in TB treatment outcomes include immunological, socio‐cultural and clinical factors [30, 34, 35, 36]. Socio‐cultural factors are complex and varied, and clinical factors are patient‐specific, highlighting the need for detailed data to determine and address the underlying causes. Patient specific data on clinical factors, for example, co‐morbidities may also be of value in identifying the underlying causes of age as a risk factor [37, 38. As a result of China's aging demographic, diabetes, which is associated with unsuccessful TB treatment outcomes, is becoming significantly more prevalent [39, 40. Although China has a policy of treating HIV patients at separate institutions, screening for and clinical management of confounders such as diabetes may be of benefit in improving TB treatment outcomes [40, 41.

For the patient population in this study, systematic and full process treatment management were associated with more favourable treatment outcomes and as such these treatment regimens are recommended where resources allow. Directly Observed Treatment, Short‐course (DOTS) continues to be key to the WHOs Stop TB Strategy [42], a component of which includes the direct observation of drug intake [43]. The significant reduction in TB prevalence that has been achieved in China is primarily attributed to the implementation of DOTS [44, 45. There is, however, debate in the literature on how much credit should be attributed to DOTS and to what extent other factors are responsible [46, 47. This raises the question of which strategies and interventions are really achieving the most resource‐effective outcomes [47].

The same is true of the conclusions drawn from this study. Are the risk factors themselves responsible for unsuccessful treatment outcomes or are confounders such as comorbidities, malnutrition, substance abuse, underlying causes of unsuccessful treatment outcomes? This ambiguity highlights the need for access to detailed data if TB control programs are going to succeed in reducing the personal and societal burden of this disease.

A limitation of this study is the lack of detailed data that would have allowed potential confounders (e.g. diabetes mellitus, substance abuse) to be interrogated. However, the large size of the patient cohort is a significant strength. Despite the large cohort, it is acknowledged that these data may not be representative of Hunan's total TB patient population. Although TB reporting is mandatory in China, there may be potential under‐reporting [48]. Patients may also seek care from traditional healers and therefore not be captured in the database [49].

## CONCLUSION

This study found that demographic (e.g. sex, age) and clinical factors (e.g. year of patient registration, illness severity, history of TB treatment and management regime) were significantly associated with unsuccessful TB treatment outcomes. The underlying causes of the demographic and clinical risk factors need to be interrogated so that effective strategies can be implemented to achieve the End TB Strategy. Consideration is required on data requirements to maximise the efficacy of TB control programmes. Both TB programmes and their associated data requirements need to evolve as the disease and confounders change over time.
